# Prevalence, antimicrobial resistance and detection of resistance genes of *Escherichia coli* and *Salmonella* spp. isolated from captive European wild boars reared in Brazil

**DOI:** 10.1007/s00203-026-05087-y

**Published:** 2026-08-01

**Authors:** Adriana Marques Faria, Itallo Conrado Sousa Araújo, Samantha Verdi Figueira, Maria Auxiliadora Andrade, Moema Pacheco Chediak Matos, Ana Maria de Souza Almeida, Guido Fontgalland Coelho Linhares

**Affiliations:** 1 Department of Preventive Veterinary Medicine, UFG, Universidade Federal de Goiás, Goiânia, Brazil; 2 Department of Animal Science, UFMG, Universidade Federal de Minas Gerais, Belo Horizonte, Brazil

**Keywords:** Antimicrobial resistance, Multidrug resistance, Virulence factors, Zoonotic bacteria, Wildlife reservoirs, One Health

## Abstract

The expansion of invasive European wild boar (*Sus scrofa scrofa*) populations in Brazil and the increasing commercialization of this species raise concerns about their role as reservoirs of zoonotic and antimicrobial-resistant bacteria. This study aimed to determine the prevalence, antimicrobial resistance profiles, and detection of virulence and resistance genes in *Escherichia coli* and *Salmonella* spp. isolated from captive wild boars. A total of 100 rectal swab samples were collected from animals raised on commercial farms in Goiás State, Brazil. One *Salmonella* isolate (1%) was detected and identified as serotype O:6,8, showing phenotypic resistance to ampicillin, amoxicillin, and sulfonamides, but no virulence or resistance genes were detected. *E. coli* was isolated from 56% of samples, with 98.2% of isolates classified as multidrug-resistant and all presenting antimicrobial resistance index values above 0.2. High resistance rates were observed for sulfonamides (96.8%), tetracycline (92.1%), amoxicillin (87.3%), ampicillin (85.7%), doxycycline (81.0%), whereas most isolates remained susceptible to ceftriaxone. Virulence genes were detected in a subset of isolates, including *tsh* (29.8%), *papC* (12.3%), and *iss* (7.0%), with some isolates presenting gene combinations associated with increased pathogenic potential. The *eae* gene was not detected, and no β-hemolytic activity was observed. These findings indicate a low occurrence of *Salmonella* but a high prevalence of multidrug-resistant and potentially pathogenic *E. coli* in captive wild boars, highlighting their potential role as reservoirs of antimicrobial resistance within a One Health context.

## Introduction

European wild boars (*Sus scrofa scrofa*), an invasive subspecies introduced into Brazil for commercial farming and hunting purposes, have rapidly expanded their distribution across all major Brazilian biomes following escapes from captivity and deliberate releases (Silva et al. 2020; Hegel et al. 2022). These animals are considered a public health concern due to their potential role as reservoirs of zoonotic pathogens and antimicrobial-resistant (AMR) bacteria. Several studies have identified *S. scrofa scrofa* as carriers of important zoonotic agents, including *Salmonella* spp., *Leptospira* spp., *Toxoplasma gondii*, *Brucella* spp., *Mycobacterium tuberculosis*, and hepatitis E virus (Bertelloni et al. 2020; Torres et al. 2020, 2021; Carraro et al. 2022). Moreover, hybridization between wild boars and domestic pigs has been increasingly reported in Brazil, particularly in southern and southeastern regions, facilitating the exchange of pathogens and resistance genes between wildlife and livestock populations (Garcia et al. 2011; Kmetiuk et al. 2023). This growing interface between wild boars, domestic animals, and humans represents a significant risk for dissemination of zoonotic diseases within a One Health framework (Kmetiuk et al. 2023).

Among enteropathogens of major zoonotic concern, *Salmonella* spp. and *Escherichia coli* are particularly relevant in wild pig populations (Faria et al. 2024). *Salmonella* has been widely reported in wild boars in Europe, with variable prevalence rates and frequent association with serotypes implicated in human outbreaks, often exhibiting multidrug resistance (Bertelloni et al. 2020). The pathogenicity of *Salmonella* ssp. is multifactorial and involves genomic elements such as pathogenicity islands, fimbrial adhesins, and flagellar structures that enable host invasion and systemic dissemination (Caleja et al. 2011). Therefore, the circulation of *Salmonella* strains harboring virulence and resistance determinants in invasive wild boar populations is of increasing concern for both veterinary and public health.

*Escherichia coli* is widely distributed in the intestinal microbiota of birds and mammals and can be disseminated through environmental matrices such as water, soil, and fomites (Kaper et al. 2004; Costa et al. 2022; Zhang et al. 2021). Certain strains are not only pathogenic but also exhibit antimicrobial resistance profiles that are concerning for One Health, being responsible for infections in both animals and humans and acting as environmental contaminants (Costa et al. 2008; Islam et al. 2021; Faria et al. 2024). Studies conducted in wild boar populations in Europe have identified extended-spectrum beta-lactamase, ESBL-/AmpC-producing *E. coli*, including strains carrying plasmid-mediated resistance genes such *blaCTX-M 1 and blaTEM*, indicating that wild boars can act as environmental reservoirs of antimicrobial resistance determinants (Costa et al. 2008). In addition, virulence-associated genes such as *papC*, *tsh* and *iss* have been reported in *E. coli* isolates from wild pigs (Faria et al. 2024), reinforcing their potential role in zoonotic transmission.

In addition, virulence-associated genes such as *papC*, *tsh* and *iss* have been reported in *E. coli* isolates from wild pigs (Faria et al. 2024), reinforcing their potential role in zoonotic transmission. The virulence-associated genes investigated in this study (*papC*, *tsh*, *iss*, and *eae*) were selected because they are among the markers most evaluated in previous studies investigating *E. coli* from wildlife and animal hosts (Ewers et al. 2005; Rodríguez-Siek et al. 2005; Faria et al. 2024). However, these genes represent only a limited subset of the extensive repertoire of virulence factors described in *E. coli*, and their presence alone does not allow assignment of isolates to specific pathotypes or prediction of pathogenicity. The *papC* gene encodes the assembly of P fimbriae, adhesins primarily associated with extraintestinal pathogenic *E. coli*. The temperature-regulated adhesin encoded by the *tsh* gene is involved in the early stages of host colonization and encodes proteins with hemagglutinating activity (Gyles et al. 2010). The *iss* gene contributes to increased serum survival by protecting *E. coli* against complement-mediated killing and phagocytosis and is frequently detected in extraintestinal pathogenic strains capable of causing septicemia (Mohamed et al. 2014).

Furthermore, environmental exposure pathways, including contaminated water sources, soil, and indirect contact with domestic livestock, may contribute to the acquisition and spread of antimicrobial resistance in wild boar populations, even in the absence of direct antimicrobial usage (Torres et al. 2020). In addition, the increasing intensification and commercialization of wild boar farming in Brazil may amplify the risk of pathogen transmission and antimicrobial resistance dissemination, particularly in systems with limited biosecurity measures (Hegel et al. 2022; Kmetiuk et al. 2023).

Despite growing evidence from European studies, data on the occurrence, antimicrobial resistance patterns, and virulence gene profiles of *Enterobacteriaceae* in farmed wild boars in Brazil remain scarce. Given the rapid expansion of captive wild boar production and their potential role as reservoirs and disseminators of zoonotic pathogens and antimicrobial resistance, further investigation is warranted. Therefore, the aim of this study was to isolate and characterize *Escherichia coli* and *Salmonella* spp. from fecal samples of farmed *S. scrofa scrofa* in the state of Goiás, Brazil, focusing on antimicrobial susceptibility profiles and the detection of virulence and resistance genes.

## Materials and methods

All procedures used in the experiments of this study received prior approval from the Animal Ethics Committee of the Universidade Federal de Goiás, Brazil (Protocol 102/2015).

### Sample collection

Rectal swab samples were collected from clinically healthy male and female European wild boars (*Sus scrofa scrofa*) reared under legal authorization at two commercial farms licensed by the Brazilian Institute of Environment and Renewable Natural Resources (IBAMA), located in Goiás State, Brazil, between September 2014 and February 2017.

The samples were obtained using sterile swabs inserted rectally and immediately placed into tubes containing 10 mL of 1% peptone water (Kasvi, Brazil). A total of 100 samples were collected (50 per farm). Each sample was obtained from a different clinically healthy animal. Sampling was performed in different enclosures within each farm, according to animal availability and safe handling conditions during the farm visits. All samples were maintained under refrigeration (2–8 °C) and transported to the laboratory within 24 h for microbiological analysis. All procedures were carried out at the Ornithopathology Laboratory, School of Veterinary and Animal Sciences, Federal University of Goiás, Brazil.

### Isolation and identification of *Salmonella* spp.

Isolation of *Salmonella* spp. was performed following the protocol described in a previous study (Faria et al. 2024), with minor adaptations. Briefly, rectal swabs were pre-enriched in 1% peptone water (Kasvi, Brazil) at 37 °C for 18–24 h, followed by selective enrichment in Selenite Cystine broth (Merck Millipore, Germany) and Rappaport–Vassiliadis broth (Merck Millipore, Germany).

Cultures were streaked onto Brilliant Green agar (Himedia, India) and Hektoen enteric agar (Kasvi, Brazil) and incubated at 37 °C for 18–24 h. Presumptive *Salmonella* colonies were subcultured on triple sugar iron (TSI) agar (Merck, Germany), and confirmation was performed using standard biochemical tests (Sobestiansky et al. 2005; ANVISA 2016).

### Isolation and identification of *Escherichia coli* and hemolysis test

Rectal swabs previously incubated in 1% peptone water at 37 °C for 18–24 h were aliquoted (~ 1 mL) and inoculated into brain heart infusion (BHI) broth (Kasvi, Brazil), followed by incubation at 37 °C for 18–24 h. Cultures were then streaked onto MacConkey agar plates for selective isolation of lactose-fermenting colonies. Because the isolation protocol targeted lactose-fermenting colonies, atypical lactose-negative *E. coli* strains may have been missed, which should be considered when interpreting the absence of certain virulence genes, such as *eae*.

Between three and five colonies showing typical *E. coli* morphology were selected from each positive sample for biochemical identification. Colonies exhibiting identical biochemical profiles were considered representative of a single isolate from that animal and one representative isolate was retained for subsequent antimicrobial susceptibility testing and molecular characterization.

Hemolytic activity was evaluated by streaking isolates onto BHI agar supplemented with 5% defibrinated sheep blood. Plates were incubated at 37 °C for 18–24 h, and hemolytic patterns were recorded.

### Antimicrobial susceptibility testing and multiple antimicrobial resistance index

Antimicrobial susceptibility of *Escherichia coli* and *Salmonella* isolates was evaluated by the disk diffusion method on Mueller–Hinton agar, following the Clinical and Laboratory Standards Institute (CLSI) guidelines. Inhibition zone diameters were interpreted according to the CLSI Performance Standards for Antimicrobial Susceptibility Testing (M100, 27th edition; CLSI 2017), using the interpretive criteria established for Enterobacteriaceae. The antimicrobial panel consisted of ampicillin (10 µg), amoxicillin (10 µg), ceftriaxone (30 µg), chloramphenicol (30 µg), tetracycline (30 µg), gentamicin (10 µg), sulfonamides (300 µg), trimethoprim–sulfamethoxazole (25 µg), and ciprofloxacin (5 µg).

The multiple antimicrobial resistance index (MAR) was calculated as the ratio between the number of antimicrobial classes to which each isolate was resistant and the total number of antimicrobial classes represented in the panel (seven). Indices > 0.2 were considered indicative of multidrug resistance (CLSI 2017). Additionally, isolates resistant to three or more antimicrobial agents from different classes were classified as multidrug-resistant (CLSI 2017).

### DNA extraction

For *E. coli*, DNA extraction was performed as follows: the isolates were inoculated in 4 mL of BHI broth and incubated at 37 °C for 18–24 h. Subsequently, 1 mL of culture was centrifuged at 13,200 rpm, the supernatant discarded, and the pellet washed with 800 µL of ultrapure water. After a second centrifugation step, the pellet was resuspended in 80 µL of ultrapure water and heated at 96 °C for 10 min. The supernatant was collected and stored at − 20 °C until PCR analysis.

For *Salmonella*, the isolates grown in Selenite Cystine broth were centrifuged (13,200 rpm for 4 min), resuspended in TE buffer, vortexed, and centrifuged again (13,200 rpm for 8 min). The pellet was resuspended in 100 µL of TE buffer, heated at 95 °C for 20 min, and stored at − 20 °C.

### Detection of *Escherichia coli* virulence genes by PCR

Genomic DNA extracted from *E. coli* isolates was subjected to PCR amplification for the detection of virulence genes *iss*, *tsh*, *papC*, and *eae*, using primers and amplification conditions (Table 1) as previously described (Ewers et al. 2005; Rodríguez-Siek et al. 2005; Yu and Kaper 1992). Only virulence-associated genes were investigated in *E. coli* isolates. Antimicrobial resistance genes were not evaluated.

**Table 1:** Primers for detection of *E. coli* virulence genes by the PCR technique

Target gene	Sequence	References
*papC*	f 5′–TGATATCACGCAGTCAGTAGC-3′ r 5′–CCGGCCATATTCACATAA-3′	Rodriguez-Siek et al. (2005)
*iss*	f 5′-ATC ACA TAG GAT TCT GCC G-3′ r 5′-CAG CGG AGT ATA GAT GCC A-3′	Ewers et al. (2005)
*tsh*	f 5′-ACT ATT CTC TGC AGG AAG TC-3′ r 5′-CTT CCG ATG TTC TGA ACG T-3′	Ewers et al. (2005)
*eae*	f 5′-AAACAGGTGAAACTGTTGCC-3′ r 5′-CTCTGCAGATTAACCTCTGC-3′	Yu and Kaper (1992)

PCR reactions were performed in a Mastercycler Personal Eppendorf^®^ thermocycler. Positive controls consisted of reference *E. coli* strains provided by the University of São Paulo, Brazil.

### Detection of *Salmonella* virulence and resistance genes by real-time PCR

Real-time PCR assays targeting the genes *spvC*, *intl1*, *sul1*, *blaTEM*, *rfbJ*, *prot6E*, *qnrA*, and *qnrB* (Table 2) were performed as previously described (Bugarel et al. 2011; Muñoz et al. 2010; Malorny et al. 2007; Dheilly et al. 2012). Amplifications were conducted using a StepOne Plus (Applied Biosystems^®^) thermocycler under the following conditions: 60 °C for 30 s, 95 °C for 10 min, followed by 40 cycles of 95 °C for 15 s and 60 °C for 1 min. The data were analyzed using StepOne Software v2.1 with a confidence level of 95%.

**Table 2:** Primers for detection of *Salmonella* virulence genes by the PCR technique

Target gene	Sequence	References
*spvC*	f 5′- AATGAACTACGAAGTGGGCG-3′ r 5′-TCAAACGATAAAACGGTTCCTC-3′ sonda FAM-ATGGTGGCGAAATGCAGAGACAGGC-BHQ	Bugarel et al. (2011)
*sul1*	f 5′-TCCTGACCCTGCGCTCTATC-3′ r 5′-TGCGCTGAGTGCATAACCA-3′ sonda ROX-ATTGCTGAGGCGGACTGCAGGC-BHQ	Bugarel et al. (2011)
*intl*	f 5′-TGGGCAGCAGCGAAGTC-3′ r 5′-TGCGTGGAGACCGAAACC-3′ sonda FAM-AGGCATTTCTGTCCTGGCTGGCG-BHQ	Bugarel et al. (2011)
*blaTEM*	f 5′-CTGGATCTCAACAGCGG-3′ r 5′-CAACACGGGATAATACCGC-3′ sonda FAM-AGATCCTTGAGAGTTTTCGCCCCG-BHQ	Bugarel et al. (2011)
*rfbJ*	Fw: 5′-GCATTTACCACATCATCTAC-3′ Rv: 5′-GCGATTAGAGCATGTATATG-3′ Sonda: FAM-TCTCTTATCTGTTCGCCTGTTGT-BHQ	Muñoz et al. (2010)
*prot6E*	Fw: 5′- ATATCGTCGTTGCTGCTTCC − 3 Rv: 5′- CATTGTTCCACCGTCACTTTG − 3′ Sonda: FAM-AGGCGCTCATCGGTCCTGCTGT-BHQ	Malorny et al. (2007)
*qnrA*	Fw: 5′- CTTCCTTGATGGGCTCAGATC − 3′ Rv: 5′- CCAGATCGGCAAAGGTTAGG − 3′ Sonda: FAM-CAGCCCCGCAGATTGACCTGTTG-BHQ	Dheilly et al. (2012)
*qnrB*	Fw: 5′ – GTAGCGCATATATCACGAATACC − 3′ Rv: 5′ – AGATCTGAACCACTGAACGTC – 3′ Sonda: FAM-ACCTGGGCACCTATCCAACGGTTT-BHQ	Dheilly et al. (2012)

The molecular methods employed for each bacterial species followed previously validated protocols available for the respective target genes; therefore, conventional PCR was used for *E. coli* and real-time PCR for *Salmonella* according to the original assay validation.

## Results

The prevalence of *Salmonella* spp. in captive European wild boars (*Sus scrofa scrofa*) was low, with only one positive isolate (1.0%) detected among the 100 rectal swab samples analyzed. The isolate was identified as serotype O:6,8 and showed phenotypic resistance to ampicillin, amoxicillin, and sulfonamides, intermediate resistance to neomycin, chloramphenicol, and tetracycline, and susceptibility to ceftriaxone, doxycycline, and sulfamethoxazole–trimethoprim. No virulence or antimicrobial resistance genes (*spvC*,* intl1*,* sul1*,* blaTEM*,* rfbJ*,* prot6E*,* qnrA*, and *qnrB*) were detected in the *Salmonella* isolate.

*Escherichia coli* was isolated from 56 of the 100 samples (56.0%). Among these, 55 isolates (98.2%) were classified as multidrug-resistant, exhibiting resistance to four or more antimicrobial agents, whereas one isolate (1.8%) showed resistance to two antimicrobial agents (Table 3). All isolates presented a MAR index greater than 0.2, indicating widespread multidrug resistance. High resistance frequencies were observed for sulfonamides (96.8%), sulfamethoxazole–trimethoprim (93.7%), tetracycline (92.1%), amoxicillin (87.3%), ampicillin (85.7%), and doxycycline (81.0%). Lower resistance frequencies were observed for neomycin (7.9%) and ceftriaxone (3.2%). A diversity of multidrug resistance profiles was observed among the *E. coli* isolates (Table 3). No isolate exhibited β-hemolytic activity.

**Table 3:** Distribution of resistance patterns to 9 antimicrobials tested in *E. coli* isolates in *S. scrofa*

Resistance rating	Numbers standard resistance	%
Multiple	*4+*	98.2
Double	*1*	1.8
Resistance profile	Resistance patterns	Number of strains
Multiple	DOX CLO TET NEO AMP AMO SUL SXT	7
Multiple	DOX TET CEF AMP AMO SUL SXT	1
Multiple	DOX CLO TET AMP AMO SUL SXT	9
Multiple	DOX TET NEO AMP AMO SUL SXT	8
Multiple	DOX CLO NEO CEF AMO SUL SXT	1
Multiple	DOX TET AMP AMO SUL SXT	20
Multiple	DOX TET AMP SUL SXT	3
Multiple	DOX CLO TET SUL SXT	1
Multiple	DOX TET NEO SUL SXT	1
Multiple	CLO TET AMP SUL SXT	2
Multiple	DOX TET AMP AMO	1
Multiple	DOX TET SUL SXT	1
Double	DOX TET	1

*AMO* Amoxicillin, *AMP* Ampicillin, *CLO* Chloramphenicol, *CEF* Ceftriaxone, *DOX* Doxycycline, *NEO* Neomycin, *SUL* Sulfonamide, *SXT* Trimethoprim–sulfamethoxazole, *TET* Tetracycline

Virulence genes were detected in a subset of isolates. The *tsh* gene was the most frequent, detected in 17 isolates (29.8%), followed by *papC* in seven isolates (12.3%) and *iss* in four isolates (7.0%). The *eae* gene was not detected in any isolate. Co-occurrence of virulence genes was observed in a small number of isolates, including *iss + tsh* (*n* = 2) and *tsh + papC* (*n* = 3) (Table 4).

**Table 4:** Positive results for virulence genes and presence of hemolysin in *E. coli* isolated from captive *S. scrofa*

Sample	iss	tsh	papC	eae
H4	**–**	**+**	**+**	**–**
H5	**–**	**–**	**–**	**–**
H6	**–**	**–**	**+**	**–**
H7	**–**	**–**	**+**	**–**
H17	**+**	**–**	**–**	**–**
H19	**–**	**+**	**–**	**-**
H21	**+**	**+**	**–**	**–**
H22	**–**	**+**	**-**	**–**
H23	**+**	**+**	**–**	**–**
H24	**–**	**+**	**–**	**–**
H26	**–**	**+**	**+**	**–**
H29	**–**	**+**	**+**	**–**
H48	**–**	**+**	**–**	**–**
H49	**–**	**+**	**–**	**–**
H50	**–**	**+**	**–**	**–**
H54	**–**	**+**	**–**	**–**
H60	**–**	**+**	**–**	**–**
H75	**–**	**-**	**+**	**–**
H76	**–**	**–**	**+**	**–**
H81	**–**	**+**	**–**	**–**
H83	**–**	**+**	**–**	**–**
H84	**–**	**+**	**–**	**–**
H85	**–**	**+**	**–**	**–**
G8	**+**	**–**	**–**	**–**

*iss* increased serum survival, *tsh* temperature-sensitive hemagglutinin, *papC* pilus-associated pyelonephritis, and *eae* enteric attachment and effacement

## Discussion

To our knowledge, this is one of the first studies in Brazil to isolate and characterize *Salmonella* spp. and *Escherichia coli* from farmed European wild boars (*Sus scrofa scrofa*), including antimicrobial resistance profiling and detection of virulence genes. Until recently, data on bacterial pathogens in captive wild boars in Brazil have been scarce (Torres et al. 2020; Kmetiuk et al. 2023). Previous studies, such as Faria et al. (2024), have demonstrated that Brazilian native peccary species (white-lipped and collared peccaries) reared in captivity may serve as reservoirs of multidrug-resistant and potentially pathogenic *E. coli*, even when raised under extensive production systems without antimicrobial use. This reinforces growing global evidence that *S. scrofa scrofa*, as an invasive species, may represent both environmental and public health risks (Bertelloni et al. 2020; Carraro et al. 2022).

The detection of only one *Salmonella* isolate suggests a low prevalence in captive wild boars under the conditions evaluated. However, this finding should be interpreted with caution. The identified serotype (O:6,8) belongs to the C2–C3 serogroup, which includes serotypes associated with poultry and swine production systems in Brazil (Rabelo et al. 2020; Pavon et al. 2022). Although none of the investigated resistance genes were detected, the phenotypic resistance observed to β-lactams and sulfonamides remains of concern. The low detection rate may be influenced by several factors, including environmental conditions, management practices, microbial competition, and intermittent bacterial shedding, which can limit the sensitivity of conventional culture methods (Hooton et al. 2011; Pavon et al. 2022).

In contrast, the extremely high frequency of multidrug-resistant *E. coli* (98.2%) is a striking finding, particularly considering that according to information provided by the farm managers, antimicrobials were not routinely administered to the wild boars. This information was based on farm management reports and was not independently verified through farm records or environmental sampling. Similar resistance levels have been reported in intensively raised domestic pigs in Brazil (Gebreyes et al. 2006; Oliveira et al. 2024), suggesting that environmental contamination may play a key role in the dissemination of antimicrobial resistance. Potential sources include contaminated water, soil, feed, and indirect contact with domestic animals or wildlife reservoirs (Martinez 2009). This finding is consistent with the hypothesis that antimicrobial resistance can be maintained and disseminated in the environment independently of direct antimicrobial use, highlighting the importance of environmental resistomes in livestock and wildlife systems (Graham et al. 2019). In addition, mobile genetic elements such as plasmids and integrons may facilitate the horizontal transfer of resistance genes between environmental bacteria and commensal *E. coli*, contributing to the persistence of multidrug resistance in these populations (Szmolka and Nagy 2013).

The antimicrobial susceptibility profile observed in this study showed high resistance rates to tetracyclines, β-lactams, and sulfonamides, which are among the most used antimicrobials in veterinary medicine. These findings are consistent with previous reports in both domestic and wild animal populations (Schwarz and Chaslus-Dancla 2001; Litterio et al. 2026), reinforcing the widespread distribution of resistance to these antimicrobial classes. On the other hand, the high susceptibility to ceftriaxone observed in this study is noteworthy. Ceftriaxone is a third-generation cephalosporin of critical importance in human medicine, and resistance to this antimicrobial is considered a major public health concern (Garcês and Pires 2023; Costa et al. 2022; Faria et al. 2024; Höcketstaller et al. 2025). The detection of even low levels of resistance to ceftriaxone should be carefully monitored due to the potential for co-selection and dissemination of resistance determinants.

Regarding virulence factors, the genes *tsh*, *papC*, and *iss* were detected at frequencies comparable to those reported in other studies involving wild and domestic animals (Faria et al. 2024). Although the presence of individual virulence genes does not necessarily indicate pathogenicity (Dionisio et al. 2023), the co-occurrence of these genes, as observed in some isolates, may enhance bacterial fitness and virulence potential. These genes are associated with mechanisms such as adhesion, serum resistance, and colonization (Faria et al. 2024), which may facilitate host infection under favorable conditions.

The absence of the *eae* gene and β-hemolytic activity suggests that the isolates were unlikely to belong to typical attaching-and-effacing pathotypes such as classical enteropathogenic *Escherichia coli* (EPEC). However, other diarrheagenic or extraintestinal pathotypes cannot be excluded because additional virulence-associated genes were not investigated (Kaper et al. 2004). The coexistence of multidrug resistance and virulence genes in *E. coli* isolates highlights the potential role of these bacteria as genetic reservoirs capable of contributing to the emergence of novel pathogenic strains through horizontal gene transfer.

The findings of this study reinforce the role of captive wild boars as reservoirs of antimicrobial-resistant and potentially pathogenic bacteria. Given the increasing expansion of wild boar farming in Brazil (Hegel et al. 2022; Silva et al. 2020), these findings suggest that antimicrobial-resistant bacteria may persist in the production environment or be introduced through multiple transmission routes, even in production systems where routine antimicrobial administration was not reported. From a One Health perspective, the interaction between wild boars, domestic animals, and humans may facilitate the circulation of antimicrobial resistance genes across ecological boundaries, increasing the risk of transmission through direct contact, environmental exposure, or the food chain (Kmetiuk et al. 2023).

The findings of this study suggest that captive European wild boars may represent a potential reservoir of antimicrobial-resistant bacteria. These results reinforce the need for further One Health surveillance integrating wildlife, livestock, humans, and the environment to better understand the epidemiology and transmission dynamics of antimicrobial resistance (Robinson et al. 2016; Hernando-Amado et al. 2019).

This study has some limitations that should be considered when interpreting the results. The samples were collected between 2014 and 2017; therefore, the findings represent the epidemiological scenario during that period and should not be interpreted as reflecting the current situation. Nevertheless, these data provide important baseline information on antimicrobial resistance and virulence characteristics of *Escherichia coli* and *Salmonella* spp. in captive European wild boars, a population for which information remains scarce in Brazil. In addition, although animals of different ages were included in the study, individual age information was not recorded. Therefore, the possible influence of age on the occurrence of *Escherichia coli* and *Salmonella* spp. could not be evaluated and should be addressed in future studies.

## Conclusion

This study demonstrated a low occurrence of *Salmonella* spp. but a high prevalence of multidrug-resistant *Escherichia coli* carrying virulence-associated genes in captive European wild boars (*Sus scrofa scrofa*) in Brazil. These findings suggest that captive wild boars may represent a potential reservoir of antimicrobial-resistant bacteria, highlighting the importance of monitoring bacterial pathogens in captive wildlife production systems. The detection of multidrug-resistant *E. coli* in animals raised without reported antimicrobial use suggests that environmental pathways may contribute to the persistence and dissemination of antimicrobial resistance. Overall, this study reinforces the need for integrated One Health surveillance involving wildlife, livestock, humans, and the environment to better understand and mitigate the spread of antimicrobial resistance.

## Data Availability

The data that support the findings of this study are available from the corresponding author upon reasonable request.
